# Relative inhibitory activities of newly developed diazabicyclooctanes, boronic acid derivatives, and penicillin-based sulfone β-lactamase inhibitors against broad-spectrum AmpC β-lactamases

**DOI:** 10.1128/aac.00775-24

**Published:** 2024-10-04

**Authors:** Christophe Le Terrier, Patrik Mlynarcik, Mustafa Sadek, Patrice Nordmann, Laurent Poirel

**Affiliations:** 1 Medical and Molecular Microbiology, Faculty of Science and Medicine, University of Fribourg, Fribourg, Switzerland; 2 Division of Intensive Care Unit, University Hospitals of Geneva, Geneva, Switzerland; 3 Department of Microbiology, Faculty of Medicine and Dentistry, Palacky University Olomouc, Olomouc, Czechia; 4 Department of Food Hygiene and Control, Faculty of Veterinary Medicine, South Valley University, Qena, Egypt; 5 Swiss National Reference Center for Emerging Antibiotic Resistance, Fribourg, Switzerland; Universita degli studi di roma La Sapienza, Rome, Italy

**Keywords:** AmpC, enmetazobactam, zidebactam, nacubactam, taniborbactam, relebactam, vaborbactam, avibactam, xeruborbactam, durlobactam, class C, β-lactamase

## Abstract

The relative inhibitory activities of diazabicyclooctanes (avibactam, relebactam, zidebactam, nacubactam, durlobactam), boronic acid derivatives (vaborbactam, taniborbactam, xeruborbactam), and penicillin-based sulfone derivative enmetazobactam were evaluated against several intrinsic and acquired class C β-lactamases. By contrast to vaborbactam and enmetazobactam, taniborbactam, xeruborbactam, and all diazabicyclooctanes demonstrated effective activities against most AmpC enzymes. Notably, durlobactam exhibited the most pronounced inhibitory effect. Interstingly, the chromosomal AmpC of *Acinetobacter baumannii* was the least sensitive enzyme to the newly developed β-lactamase inhibitors.

## INTRODUCTION

Resistance to β-lactam antibiotics in Gram-negative pathogens is mainly driven by an enzymatic mechanism, such as the production of β-lactamases (1, 2). Each pathogen can acquire β-lactamase genes by horizontal transfer related to genetic mobile elements and, therefore, produce several different enzymes, eventually leading to multidrug resistance. These β-lactamases have been classified according to their amino-acid sequence (the so-called Ambler classification), resulting in four main groups (3, 4). Class C β-lactamases, also known as AmpC enzymes, are commonly identified among Gram-negative bacteria, being either intrinsic to the respective species and in those cases chromosomally encoded (e.g., in *Pseudomonas aeruginosa*, *A. baumannii*, *Enterobacter cloacae*, *Serratia marcescens*) or acquired by horizontal gene transfer via transposons and insertion sequence elements but mostly plasmid encoded (5). AmpC enzymes hydrolyze penicillins, first-generation cephalosporins (e.g., cephalothin), and cephamycins (e.g., cefoxitin), and are not significantly inhibited by clavulanic acid (CLA), sulbactam, and tazobactam (TAZ) (5, 6). When overproduced, as a result either of a dysregulation of the gene expression through defective AmpR-like regulators or of a plasmid location and, therefore, high-copy number, they also significantly hydrolyze broad-spectrum cephalosporins such as ceftriaxone, cefotaxime, and ceftazidime (CAZ). Worryingly, the most acquired AmpC enzymes hydrolyze first-line β-lactams that may be used in community infections, such as cefotaxime or ceftriaxone. Nevertheless, broad-spectrum β-lactams such as cefepime, cefiderocol, and carbapenems are usually not significantly affected by the hydrolytic activities of AmpC enzymes (5, 6).

Recently, the development of new β-lactamase inhibitors belonging to different classes, such as the diazabicyclooctane (DBO) avibactam (AVI), relebactam (REL), zidebactam (ZID), nacubactam (NAC), and durlobactam (DUR); the boronic acid derivatives (BAD) vaborbactam (VAB), taniborbactam (TAN), and xeruborbactam (XER); and the penicillin-based sulfone derivative enmetazobactam (EMT) has significantly enlarged the antibiotic pipeline by promoting the development of novel β-lactam/β-lactamase inhibitor (BL/BLI) combinations. The currently commercialized combinations are ceftazidime-avibactam, imipenem-relebactam, sulbactam-durlobactam, and meropenem-vaborbactam, and some others are currently under clinical evaluations including aztreonam-avibactam, cefepime-taniborbactam, cefepime-enmetazobactam, meropenem-nacubactam, and meropenem-xeruborbactam (7–10). However, many unusual therapeutic associations combining a β-lactamase inhibitor with a β-lactam partner might be considered when facing difficult-to-treat pathogens (10, 11).

The aim of our study was therefore to precisely evaluate the relative inhibitory activities of the recently developed and abovementioned β-lactamase inhibitors, including DBOs, BAD, and the EMT molecule, using isogenic *Escherichia coli* recombinant strains producing a wide range of AmpC β-lactamases.

In order to assess the relative inhibitory activities of those BLI against AmpC β-lactamases, the corresponding *bla*_ampC_ genes without the original promoter sequences were amplified by PCR with specific primers, and corresponding amplicons were cloned into plasmid pUCp24, as described previously (12, 13). Hence, all these genes were expressed under the control of the same promoter sequences, and relative comparisons were meaningful. Cultures of *E. coli* TOP10 harboring recombinant plasmids and therefore producing the different AmpC tested were grown overnight at 37°C in Luria-Bertani agar medium containing amoxicillin (AMX) (50 µg/mL). The activity of the following acquired class C β-lactamases could therefore be evaluated, namely, ACC-1 (originally from *Hafnia* spp.), FOX-5 (from *Aeromonas allosaccharophila*), CMY-2 and CMY-42 (from *Citrobacter* spp.), DHA-1 (from *Morganella morganii*), ACT-7 and ACT-17 (from *Enterobacter asburiae*), MIR-17 (from *Enterobacter roggenkampii*), MOX-2 (from *Aeromonas caviae*), and LAT-1 (from *Citrobacter portucalensis)* (5). In addition, some chromosomally encoded AmpC enzymes were added in the evaluation, including Ear-1 from *Klebsiella aerogenes*; SRT-2 from *Serratia marcescens*; PDC-1 (narrow-spectrum cephalosporinase), PDC-5 (extended-spectrum cephalosporinase), and PDC-382 (variant harboring the T96I substitution, involved in ceftolozane-tazobactam and ceftazidime-avibactam resistance) of *P. aeruginosa* (14, 15); ADC-50 from *A. baumannii* CIP7010 (16); and YRC-1 from *Yersinia ruckeri* (17). Altogether, these enzymes were chosen as representatives of clinically relevant class C β-lactamases being sources of intrinsic or acquired resistance to broad-spectrum β-lactams among most of the commonly identified Gram-negative bacteria in clinical practice.

MICs were determined in triplicate by broth microdilution (BMD) for CAZ and AMX, as well as their combinations with each β-lactamase inhibitor. CAZ, AMX, and CLA were purchased from Sigma-Aldrich (St. Louis, MO, USA). All others inhibitors (TAZ HY-W009168, AVI HY-14879, REL HY-16752, VAB HY-19930, NAC HY-109008, ZID HY-120859, TAN HY-109124, XER HY-136072, DUR HY-117974, EMT HY-103095) were purchased from MedChem Express (Luzern, Switzerland). The concentration of each β-lactamase inhibitor was chosen as the concentration used in the respective BL/BLI combination under development or commercially available, according to EUCAST or CLSI (18, 19). Hence, CLA was used at 2 µg/mL; TAZ, AVI, REL, NAC, ZID, DUR, and TAN at 4 µg/mL; and VAB, XER, and EMT at 8 µg/mL (9, 13, 17, 18). BMD assays were performed in cation-adjusted Mueller-Hinton (MH) broth (Bio-Rad, Marnes-la-Coquette, France) for all antibiotics or antibiotic combinations listed above, according to the EUCAST guidelines (20). Reference strains *E. coli* ATCC 25922, *E. coli* NCTC 13353, *E. coli* ATCC 35218, *Klebsiella pneumoniae* ATCC 700603, *K. pneumoniae* ATCC BAA-2814, *P. aeruginosa* ATCC 27853, and *A. baumannii* NCTC 13304 were used as quality control for all BLI according to EUCAST or CLSI (18, 19, 21), and all MIC values are provided in Table S1. The mean 50% inhibitory concentrations (IC_50_) of all BLI were determined for all AmpC enzymes after performing IC_50_ measurements in triplicate, using crude extracts of cultures of *E. coli* TOP10 recombinant strains, respectively, producing the different β-lactamases to be tested, as previously published (13).

The results obtained for recombinant AmpC-producing *E. coli* strains revealed a series of interesting features (Table 1). Firstly, all MIC values obtained for CAZ for AmpC producers were at least fourfold dilution higher than the MIC value observed for *E. coli* TOP10, with the exception of the MOX-2 and PDC-like recombinant strains. As expected, the combination of CAZ with the “old” inhibitors, CLA and TAZ, did not result in a significant reduction in the MIC values of all AmpC-producing *E. coli*. Interestingly, similar results were observed with the addition of the novel EMT, which highlights the inefficiency of this inhibitor against class C β-lactamase.

**TABLE 1:** MICs of β-lactams for AmpC-producing *E. coli* TOP10 recombinant strains^*a*^

Strain (β-lactamase produced)	Natural (N) or acquired (A) Ambler class C	MICs (µg/mL)^*b*^											
		Ceftazidime											
			Old inhibitors		Diazabicyclooctane					Boronate acid derivative			Penicillin sulfone
		**Alone**	**+CLA**	**+TZB**	**+AVI**	**+REL**	**+NAC**	**+ZID**	**+DUR**	**+VAB**	**+TAN**	**+XER**	**+EMT**
*E. coli* ATCC 25922	-	0.06	0.06	0.06	0.06	0.06	≤0.06	≤0.06	≤0.06	0.06	0.06	0.06	0.06
*E. coli* TOP10	-	0.25	0.25	0.25	0.125	0.25	≤0.06	≤0.06	≤0.06	0.25	0.125	0.125	0.25
*E. coli* ACC-1	A	32	**32**	**16**	0.5	0.5	0.125	≤0.06	≤0.06	4	0.125	0.125	**32**
*E. coli* FOX-5	A	8	**8**	**8**	1	1	0.125	≤0.06	≤0.06	**4**	0.125	0.125	**8**
*E. coli* ACT-7	A	64	**64**	**64**	0.25	0.5	0.125	≤0.06	≤0.06	1	0.125	0.125	**64**
*E. coli* ACT-17	A	32	**32**	**32**	0.25	0.5	0.125	≤0.06	≤0.06	0.5	0.125	0.125	**32**
*E. coli* CMY-2	A	>256	**>128**	**>128**	1	2	0.125	≤0.06	≤0.06	16	0.25	0.25	**>128**
*E. coli* CMY-42	A	>256	**>128**	**>128**	2	2	0.125	≤0.06	≤0.06	32	0.125	0.125	**>128**
*E. coli* DHA-1	A	128	**128**	**128**	0.25	1	0.125	≤0.06	≤0.06	4	0.125	0.125	**64**
*E. coli* LAT-1	A	64	**64**	**32**	0.25	1	0.125	≤0.06	≤0.06	2	0.125	0.125	**32**
*E. coli* MOX-2	A	0.5	**0.5**	**0.5**	0.25	0.25	≤0.06	≤0.06	≤0.06	0.25	0.125	0.125	**0.5**
*E. coli* MIR-17	A	4	**4**	**2**	0.25	0.25	0.125	≤0.06	≤0.06	0.25	0.125	0.125	**4**
*E. coli* Ear-1	N	>256	**>128**	**>128**	0.5	0.5	≤0.06	≤0.06	≤0.06	2	1	0.25	64
*E. coli* SRT-2	N	4	**4**	**2**	0.25	0.5	≤0.06	≤0.06	≤0.06	0.25	0.125	0.125	**4**
*E. coli* PDC-1	N	0.5	**0.5**	**0.5**	0.25	0.25	0.125	≤0.06	≤0.06	**0.5**	0.125	0.125	**0.5**
*E. coli* PDC-5	N	1	**1**	**1**	0.125	0.25	0.125	≤0.06	≤0.06	**0.5**	0.25	0.125	**1**
*E. coli* PDC-382	N	2	**2**	**1**	0.5	0.5	≤0.06	≤0.06	≤0.06	**2**	**1**	0.25	**2**
*E. coli* ADC-50	N	64	**64**	**64**	2	0.5	0.125	≤0.06	≤0.06	**64**	**32**	0.125	**64**
*E. coli* YRC-1	N	8	**8**	**8**	0.125	0.125	0.125	≤0.06	≤0.06	0.5	0.125	0.125	**8**
		**Amoxicillin**											
		**Alone**	**+CLA**	**+TZB**	**+AVI**	**+REL**	**+NAC**	**+ZID**	**+DUR**	**+VAB**	**+TAN**	**+XER**	**+EMT**
*E. coli* ATCC 25922	-	4	4	4	4	4	≤0.25	≤0.25	≤0.25	4	4	≤0.25	4
*E. coli* TOP10	-	1	1	1	0.5	0.5	≤0.25	≤0.25	≤0.25	0.5	0.5	≤0.25	1
*E. coli* ACC-1	A	32	**32**	**16**	0.5	0.5	≤0.25	≤0.25	≤0.25	4	0.5	≤0.25	**16**
*E. coli* FOX-5	A	8	**8**	**8**	1	1	≤0.25	≤0.25	≤0.25	**4**	0.5	≤0.25	**8**
*E. coli* ACT-7	A	1,024	**1,024**	**512**	2	4	≤0.25	≤0.25	≤0.25	32	8	2	**512**
*E. coli* ACT-17	A	512	**512**	**256**	1	2	≤0.25	≤0.25	≤0.25	16	4	≤0.25	**256**
*E. coli* CMY-2	A	>1,024	**>1,024**	**>512**	8	8	≤0.25	≤0.25	≤0.25	128	16	2	**>512**
*E. coli* CMY-42	A	1,024	**1,024**	**512**	2	4	≤0.25	≤0.25	≤0.25	128	8	1	**512**
*E. coli* DHA-1	A	1,024	**1,024**	**64**	1	8	≤0.25	≤0.25	≤0.25	64	8	1	**64**
*E. coli* LAT-1	A	512	**512**	**64**	0.5	1	≤0.25	≤0.25	≤0.25	16	2	≤0.25	**256**
*E. coli* MOX-2	A	8	**8**	**8**	0.5	0.5	≤0.25	≤0.25	≤0.25	0.5	0.5	≤0.25	**8**
*E. coli* MIR-17	A	128	**128**	**128**	0.5	0.5	≤0.25	≤0.25	≤0.25	2	1	≤0.25	**128**
*E. coli* Ear-1	N	>1,024	**>1,024**	**>1,024**	32	32	≤0.25	≤0.25	≤0.25	256	128	4	**>1,024**
*E. coli* SRT-2	N	128	**128**	**64**	8	16	≤0.25	≤0.25	≤0.25	32	2	≤0.25	**128**
*E. coli* PDC-1	N	16	**16**	**8**	0.5	0.5	≤0.25	≤0.25	≤0.25	2	0.5	≤0.25	**16**
*E. coli* PDC-5	N	16	**16**	**8**	0.5	0.5	≤0.25	≤0.25	≤0.25	4	0.5	≤0.25	**16**
*E. coli* PDC-382	N	16	**16**	**8**	2	2	≤0.25	≤0.25	≤0.25	8	2	≤0.25	**16**
*E. coli* ADC-50	N	512	**512**	**512**	16	8	≤0.25	≤0.25	≤0.25	**512**	**256**	≤0.25	**256**
*E. coli* YRC-1	N	512	**512**	**512**	1	2	≤0.25	≤0.25	≤0.25	64	8	≤0.25	**256**

^*a*^ MIC values in bold are those corresponding to less than a twofold change dilution of the MIC value of the β-lactam alone when testing the corresponding AmpC-producing *E. coli* TOP10. Shaded MIC values are those corresponding to a significantly decreased MIC value compared to the MIC value of the β-lactam alone when testing the corresponding AmpC-producing *E. coli* TOP10, defined as a MIC value between twofold and fivefold change dilution compared to the corresponding AmpC-producing *E. coli* TOP10. "-", no β-lactamase produced.

^*b*^ Data of minimal inhibitory concentrations by broth microdilution; CAZ, ceftazidime; CAZ-AC, ceftazidime-clavulanic acid; CAZ-TZB, ceftazidime-tazobactam; CAZ-AVI, ceftazidime-avibactam; CAZ-REL, ceftazidime-relebactam; CAZ-VAB, ceftazidime-vaborbactam; CAZ-NAC, ceftazidime-nacubactam; CAZ-ZID, ceftazidime-zidebactam; CAZ-DURL, ceftazidime-durlobactam; CAZ-TAN, ceftazidime-taniborbactam; CAZ-EMT, ceftazidime-enmetazobactam; CAZ-XER, ceftazidime-xeruborbactam. The β-lactamase inhibitors, clavulanic acid was used at fixed concentrations of 2 µg/mL; tazobactam, avibactam, relebactam, nacubactam, zidebactam, taniborbactam, and durlobactam were used at fixed concentrations of 4 µg/mL; whereas xeruborbactam, vaborbactam and enmetazobactam were used at 8 µg/mL.

With regard to the activity of the DBOs, the supplementation of AVI resulted in a decrease in MIC values by at least fivefold dilution for most recombinant strains, with the exception of the FOX-5, PDC-382, and ADC-50 producers for which the reduction was lower (Table 1). The supplementation of CAZ with other DBOs, namely, NAC, ZID, and DUR, resulted in very low MIC values. Considering that the MIC values of NAC, ZID, and DUR alone were determined at 4, 0.125, and 0.125 µg/mL, respectively, when testing *E. coli* TOP 10, those very low MIC values may reflect the strong direct antibacterial activities of those BLI against *E. coli* (9–11). Therefore, this intrinsic antibacterial activity of the DBOs limited the analysis of the relative inhibitory activity of those BLIs by the susceptibility testing approach. In accordance with results obtained with DBO molecules, combinations including TAN and XER reduced all MIC values for all recombinant strains. XER is also known to possess a moderate antibacterial activity against *E. coli* by targeting multiple penicillin-binding proteins (22). In a previous study, the MIC value of XER alone was found to be above 16 µg/mL for the *E. coli* TOP10 (13). This antibacterial activity may also limit the specific analysis of the inhibitory activity of this BLI by analyzing the MIC values reduction.

Although all AmpCs were significantly inhibited by TAN, the ADC-50 β-lactamase (cAmpC of *A. baumannii*) was found to be resistant to this inhibitor.

On the other hand, when testing CAZ with VAB, only a slight decrease in MIC values was observed for the majority of the recombinant strains, and even no significant impact was observed with VAB-based combinations when testing FOX-5, PDC-382, and ADC-50 producers.

Then, the IC_50_ values were determined for each BLI, leading to results that were overall consistent with the results of the susceptibility testing, as illustrated in Table 2. Firstly, high IC_50_ values were obtained against most AmpC enzymes when testing old inhibitors, CLA and TAZ, as expected.

**TABLE 2:** Determination of the 50% inhibitory concentration (IC_50_) for β-lactamase inhibitors against AmpC enzymes^*a*^

	IC_50_ (µM)										
	Old inhibitors		Diazabicyclooctane					Boronate acid derivative			Penicillin sulfone derivative
Enzyme	Clavulanic acid (CLA)	Tazobactam (TAZ)	Avibactam (AVI)	Relebactam (REL)	Nacubactam (NAC)	Zidebactam (ZID)	Durlobactam (DUR)	Vaborbactam (VAB)	Taniborbactam (TAN)	Xeruborbactam (XER)	Enmetazobactam (EMT)
ACC-1	**>100**	2.1	0.25	0.58	0.45	0.09	0.005	3.6	0.18	0.09	**39**
FOX-5	**>100**	**23**	1	2.0	5.3	0.99	0.022	**17**	0.16	0.23	**>100**
ACT-7	**>100**	**23**	0.8	0.71	0.37	0.12	0.004	3.6	0.19	0.29	**43**
ACT-17	**>100**	**32**	0.9	0.33	0.29	0.14	0.003	2.6	0.21	0.2	**62**
CMY-2	**>100**	**20**	0.61	0.59	0.42	0.12	0.004	5.3	0.18	0.28	**40**
CMY-42	**>100**	**24**	0.65	1.1	1.7	0.17	0.011	**13**	0.68	0.43	**64**
DHA-1	**>100**	1.3	0.11	0.85	0.50	0.06	0.004	3.1	0.12	0.16	**26**
LAT-1	**>100**	7.3	0.4	0.23	0.29	0.005	0.005	1.2	0.23	0.34	**97**
MOX-2	**>100**	**>100**	5.6	2.4	4.1	2.7	0.055	1.9	0.32	0.26	**>100**
MIR-17	**>100**	**83**	1	1.3	0.6	0.11	0.005	1.1	0.03	0.31	**>100**
Ear-1	**>100**	**72**	0.88	0.89	0.28	0.12	0.006	0.84	0.11	0.22	**91**
SRT-1	**>100**	**48**	6.7	**37**	**70**	**37**	0.022	6.9	0.06	0.22	**>100**
PDC-1	**>100**	**15**	1.4	3.0	5.1	0.42	0.009	**11**	1.1	0.67	**>100**
PDC-5	**>100**	**18**	0.86	1.9	1.2	0.09	0.013	**37**	0.51	0.66	**>100**
PDC-382	**>100**	**11**	**11**	**23**	**34**	1.9	0.1	**54**	5.0	0.32	**>100**
ADC-50	**>100**	**>100**	**61**	**13**	**>100**	5.5	0.17	**>100**	**26**	5.4	**>100**
YRC-1	**>100**	**>100**	2.1	1.9	0.13	0.09	0.005	2.0	0.1	0.96	**>100**

^*a*^ The shaded values represent IC_50_ values measured between 1 and 10 µM, while the bold values indicate measurements above 10 µM.

Our results also showed that DUR exhibited the greatest potency among all tested BLIs. Hence, DUR possessed the widest spectrum to inhibit all chromosomally encoded and acquired AmpC β-lactamases included in this study, as evidenced by the IC_50_ values being at least 10-fold lower than those observed for other BLIs. Similarly, ZID also showed excellent performance in terms of spectrum of inhibition and interestingly also possessed an intrinsic antibacterial activity, enhancing its overall efficacy. On the other hand, even if the respective IC_50_ values suggested lower potency as BLIs compared to the previously mentioned molecules, AVI, REL, and NAC also showed significant inhibitory activities against most AmpC enzymes, with the exception of the chromosomally encoded PDC-382 of *P. aeruginosa* and ADC-50 of *A. baumannii*.

When considering BLI belonging to the BAD family, the IC_50_ values obtained for XER and TAN were also very low, being in the nanomolar (nM) range for the majority of enzymes, with the exception of ADC-50, which is far less sensitive to the inhibitory action of those two molecules.

When testing VAB, variable IC_50_ values were obtained, with all values being measured in the micromolar (µM) range. These values were significantly higher than those observed for all BLIs belonging to the DBOs or BAD families, highlighting the relatively poor inhibitory activity of VAB against class C β-lactamases. It is noteworthy that, similar to the results observed with TAZ, the penicillin-based sulfone derivative, EMT, did not show any significant activity against all AmpC enzymes tested here.

Regarding the overall sensitivity of the enzymes for the newly developed BLIs, the intrinsic ADC-50 of *A. baumannii* exhibited the lowest sensitivity to the inhibitors. In contrast to the other BLIs, which exhibited significant higher values against this enzyme, only DUR exhibited a very low IC_50_ value (0.17 nM). The values of ZID and XER were both determined at 5 µM, whereas the values of REL and AVI were found to be above 13 µM. Finally, ADC-50 was not significantly inhibited by NAC, VAB, EMT, and TAN. Interestingly, the PDC-382 β-lactamase is less sensitive to all DBOs than PDC-1 and PDC-5, as illustrated by the MIC and IC_50_ values in Tables 1 and 2. This finding indicates that the amino-acid substitution T96I in the PDC protein sequence affects not only avibactam, as previously reported (23), but also all DBOs.

Noteworthy, it is important to underscore that this study focused only on the interaction between AmpC and the recently developed BLI. In a clinical context, the production level of AmpC (mainly cAmpC) may vary among Gram-negative bacteria, and therefore plays a significant role in the *in vitro* activity of BL/BLI combinations. Furthermore, clinical strains can simultaneously produce numerous other β-lactamases and exhibit permeability defects for instance.

In addition to AmpC, the newly developed BLI also exhibits a broad spectrum of activity, encompassing the majority of class A, class B for TAN and XER, and a subset of class D for AVI, ZID, DUR, TAN, and XER (7–9, 13, 22, 24). Moreover, some of these BLIs demonstrate direct antibacterial activity (7–11, 13, 22, 24, 25). Furthermore, these BLIs are invariably combined with their β-lactam partner, which can evade the hydrolysis activity of the enzyme and thereby extending the spectrum of the combination BL/BLI. As a result, the extrapolation of our data to a clinical context is limited by these factors. For instance, although VAB is not effective against the majority of AmpC, the combination of meropenem-vaborbactam remains highly effective against AmpC-producing *Enterobacterales*, given that meropenem is not hydrolyzed by AmpC enzymes (7). Cefepime-zidebactam is also highly effective against NDM-producing *E. coli*, whereas ZID does not inhibit class B β-lactamases but has a strong direct antibacterial activity against *E. coli* (25).

In conclusion, our study highlighted the remarkable efficacy of all DBOs, as well as TAN and XER (DAB family of inhibitors), in inhibiting a wide range (acquired and intrinsic) AmpC enzymes. We showed that DUR was the most effective BLI against class C β-lactamases, by contrast to VAB and EMT that exhibited poor activities.

## SUPPLEMENTAL MATERIAL

Table S1 aac.00775-24-s0001.pdf Susceptibility testing of all QC strains used in the study.

ASM does not own the copyrights to Supplemental Material that may be linked to, or accessed through, an article. The authors have granted ASM a non-exclusive, world-wide license to publish the Supplemental Material files. Please contact the corresponding author directly for reuse.

## References

[1] Blair JMA, Webber MA, Baylay AJ, Ogbolu DO, Piddock LJV. 2015. Molecular mechanisms of antibiotic resistance. Nat Rev Microbiol 13:42–51. doi:10.1038/nrmicro3380 25435309

[2] Poirel L, Madec JY, Lupo A, Schink AK, Kieffer N, Nordmann P, Schwarz S. 2018. Antimicrobial resistance in Escherichia coli. Microbiol Spectr 6. doi:10.1128/microbiolspec.ARBA-0026-2017 PMC11633601 30003866

[3] Ambler RP. 1980. The structure of beta-lactamases. Philos Trans R Soc Lond B Biol Sci 289:321–331. doi:10.1098/rstb.1980.0049 6109327

[4] Nordmann P, Poirel L. 2002. Emerging carbapenemases in Gram-negative aerobes. Clin Microbiol Infect 8:321–331. doi:10.1046/j.1469-0691.2002.00401.x 12084099

[5] Philippon A, Arlet G, Labia R, Iorga BI. 2022. Class C β-lactamases: molecular characteristics. Clin Microbiol Rev 35:e0015021. doi:10.1128/cmr.00150-21 35435729 PMC9491196

[6] Jacoby GA. 2009. AmpC beta-lactamases. Clin Microbiol Rev 22:161–182. doi:10.1128/CMR.00036-08 19136439 PMC2620637

[7] Yahav D, Giske CG, Grāmatniece A, Abodakpi H, Tam VH, Leibovici L. 2020. New β-lactam-β-lactamase inhibitor combinations. Clin Microbiol Rev 34:e00115-20. doi:10.1128/CMR.00115-20 PMC7667665 33177185

[8] Theuretzbacher U. 2023. Evaluating the innovative potential of the global antibacterial pipeline. Clin Microbiol Infect:S1198-743X(23)00490-1. doi:10.1016/j.cmi.2023.09.024 37805036

[9] Le Terrier C, Nordmann P, Freret C, Seigneur M, Poirel L. 2023. Impact of acquired broad spectrum β-lactamases on susceptibility to novel combinations made of β-lactams (aztreonam, cefepime, meropenem, and imipenem) and novel β-lactamase inhibitors in Escherichia coli and Pseudomonas aeruginosa. Antimicrob Agents Chemother 67:e00339-23. doi:10.1128/aac.00339-23 37255469 PMC10353362

[10] Le Terrier C, Freire S, Nordmann P, Poirel L. 2024. Multidrug-resistant Gram-negative clinical isolates with reduced susceptibility/resistance to cefiderocol: which are the best present and future therapeutic alternatives? Eur J Clin Microbiol Infect Dis 43:339–354. doi:10.1007/s10096-023-04732-4 38095831 PMC10821827

[11] Le Terrier C, Nordmann P, Poirel L. 2022. In vitro activity of aztreonam in combination with newly developed β-lactamase inhibitors against MDR Enterobacterales and Pseudomonas aeruginosa producing metallo-β-lactamases. J Antimicrob Chemother 78:101–107. doi:10.1093/jac/dkac360 36308322

[12] Le Terrier C, Nordmann P, Buchs C, Poirel L. 2024. Effect of modification of penicillin-binding protein 3 on susceptibility to ceftazidime-avibactam, imipenem-relebactam, meropenem-vaborbactam, aztreonam-avibactam, cefepime-taniborbactam, and cefiderocol of Escherichia coli strains producing broad-spectrum β-lactamases. Antimicrob Agents Chemother 68:e01548-23. doi:10.1128/aac.01548-23 38415988 PMC10989025

[13] Le Terrier C, Freire S, Viguier C, Findlay J, Nordmann P, Poirel L. 2024. Relative inhibitory activities of the broad-spectrum β-lactamase inhibitor xeruborbactam in comparison with taniborbactam against metallo-β-lactamases produced in Escherichia coli and Pseudomonas aeruginosa. Antimicrob Agents Chemother:e0157023. doi:10.1128/aac.01570-23 38727224 PMC11620488

[14] Rodríguez-Martínez JM, Poirel L, Nordmann P. 2009. Extended-spectrum cephalosporinases in Pseudomonas aeruginosa. Antimicrob Agents Chemother 53:1766–1771. doi:10.1128/AAC.01410-08 19258272 PMC2681535

[15] Fraile-Ribot PA, Cabot G, Mulet X, Periañez L, Martín-Pena ML, Juan C, Pérez JL, Oliver A. 2018. Mechanisms leading to in vivo ceftolozane/tazobactam resistance development during the treatment of infections caused by MDR Pseudomonas aeruginosa. J Antimicrob Chemother 73:658–663. doi:10.1093/jac/dkx424 29149337

[16] Mammeri H, Poirel L, Mangeney N, Nordmann P. 2003. Chromosomal integration of a cephalosporinase gene from Acinetobacter baumannii into Oligella urethralis as a source of acquired resistance to beta-lactams. Antimicrob Agents Chemother 47:1536–1542. doi:10.1128/AAC.47.5.1536-1542.2003 12709319 PMC153344

[17] Mammeri H, Poirel L, Nazik H, Nordmann P. 2006. Cloning and functional characterization of the ambler class C beta-lactamase of Yersinia ruckeri. FEMS Microbiol Lett 257:57–62. doi:10.1111/j.1574-6968.2006.00148.x 16553832

[18] EUCAST. 2024. Breakpoint tables for interpretation of MICs and zone diameters. Available from: https://www.eucast.org/fileadmin/src/media/PDFs/EUCAST_files/Breakpoint_tables/v_14.0_Breakpoint_Tables.pdf

[19] Clinical and Laboratory Standards Institute. 2023. Performance standards for antimicrobial susceptibility testing. In CLSI M100, 33rd ed. Clinical and Laboratory Standards Institute, Wayne, PA.

[20] EUCAST. 2024. MIC determination of non-fastidious and fastidious organisms. Available from: https://www.eucast.org/ast_of_bacteria/mic_determination

[21] EUCAST. 2024. QC tables 14.0. https://www.eucast.org/ ast_of_bacteria/quality_control.

[22] Sun D, Tsivkovski R, Pogliano J, Tsunemoto H, Nelson K, Rubio-Aparicio D, Lomovskaya O. 2022. Intrinsic antibacterial activity of xeruborbactam in vitro: assessing spectrum and mode of action. Antimicrob Agents Chemother 66:e00879-22. doi:10.1128/aac.00879-22 36102663 PMC9578396

[23] Slater CL, Winogrodzki J, Fraile-Ribot PA, Oliver A, Khajehpour M, Mark BL. 2020. Adding insult to injury: mechanistic basis for how AmpC mutations allow Pseudomonas aeruginosa to accelerate cephalosporin hydrolysis and evade avibactam. Antimicrob Agents Chemother 64:e00894-20. doi:10.1128/AAC.00894-20 32660987 PMC7449160

[24] Le Terrier C, Viguier C, Nordmann P, Vila AJ, Poirel L. 2024. Relative inhibitory activities of the broad-spectrum β-lactamase inhibitor taniborbactam against metallo-β-lactamases. Antimicrob Agents Chemother 68:e0099123. doi:10.1128/aac.00991-23 38047644 PMC10848752

[25] Le Terrier C, Nordmann P, Sadek M, Poirel L. 2023. In vitro activity of cefepime/zidebactam and cefepime/taniborbactam against aztreonam/avibactam-resistant NDM-like-producing Escherichia coli clinical isolates. J Antimicrob Chemother 78:1191–1194. doi:10.1093/jac/dkad061 36921067 PMC10154122

