# Supplementary material for: Relative inhibitory activities of newly developed diazabicyclooctanes, boronic acid derivatives, and penicillin-based sulfone β-lactamase inhibitors against broad-spectrum AmpC β-lactamases
Source: Antimicrob Agents Chemother. 2024 Oct 4;68(11):e00775-24. doi: 10.1128/aac.00775-24 (PMC11539244; doi:10.1128/aac.00775-24)
Supplement: Table S1 — Susceptibility testing of all QC strains used in the study. [file aac.00775-24-s0001.pdf]

Table S1. Susceptibility testing of all QC strains used in the study.

QC Strain	Ambler class	Minimal Inhibitory Concentrations (µg/ml) [a]											
		CAZ	AMC	PTZ	CZA	I/R	MVB	MER-NAC 1:1	FEP-ZID 1:1	SUL-DUR	FEP-TAN	FEP-ENM	MER-XER
Escherichia coli ATCC 25922	-	0.06 *(0.06-0.5)*[b]	-	4	-	0.06	0.06	0.03	0.03	-	-	0.06	-
Escherichia coli NCTC 13353	CTX-M-15, OXA-1	256	-	-	0.5	-	-	-	0.125	-	0.125 *(0.125-1)*[c]	0.125 *(0.03-0.125)*[c]	-
Escherichia coli ATCC 35218	TEM-1	-	8 *(4-32)*[b]	1 *(0.5-2)*[b]	-	0.06	-	-	-	-	0.06	-	-
Klebsiella pneumoniae ATCC 700603	SHV-18, OXA-2	64	8 *(4-16)*[c]	16 *(8-32)*[c]	1 *(0.25-2)*[b]	0.125	-	-	-	-	-	0.25	-
Klebsiella pneumoniae ATCC BAA-2814	KPC-3, SHV-11, TEM-1	-	-	-	2	0.125 *(0.06-0.5)*[b]	0.25 *(0.125-0.5)*[b]	0.5 *(0.5-2)*[c]	-	-	1	-	0.06 *(0.015-0.06)*[c]
Pseudomonas aeruginosa ATCC 27853	Inducible AmpC	2 *(1-4)*[b]	-	4	1	0.25	0.25	0.5	-	-	-	0.5	0.25
Acinetobacter baumannii NCTC 13304	OXA-27	-	-	-	-	-	-	-	4 *(4-16)*[c]	0.5 *(0.5-2)*[c]	-	-	-

[a] Data of minimal inhibitory concentrations by broth microdilution; CAZ, ceftazidime; AMC, amoxicillin-clavulanic acid; PTZ, piperacillin-tazobactam; CZA, ceftazidime-avibactam; I/R, imipenem-relebactam; MVB, meropenem-vaborbactam; MER-NAC 1:1, meropenem-nacubactam; FEP-ZID 1:1, cefepime-zidebactam at 1:1 ratio; SUL-DUR, sulbactam-durlobactam; FEP-TAN, cefepime-taniborbactam; FEP-ENM, cefepime-enmetazobactam; MER-XER, meropenem-xeruborbactam. The ß-lactamase inhibitors, clavulanic acid, tazobactam, avibactam, relebactam, taniborbactam, durlobactam were used at fixed concentrations at 4 µg/ml, while vaborbactam, xeruborbactam and enmetazobactam were used at 8mg/L.[b] MIC range of the respective QC strain according to EUCAST.[c] MIC range of the respective QC strain according to CLSI.
